# Skin histology and its role in heat dissipation in three pinniped species

**DOI:** 10.1186/1751-0147-54-46

**Published:** 2012-08-13

**Authors:** Wael A Khamas, Hrvoje Smodlaka, Jessica Leach-Robinson, Lauren Palmer

**Affiliations:** 1 College of Veterinary Medicine, Western University of Health Sciences, 309 E. Second Street, Pomona, CA 91766-1854, USA; 2 The Marine Mammal Care Center Fort MacArthur, 3601S. Gaffey St., San Pedro, CA 90731, USA

**Keywords:** Skin, Histology, Thermography, Pinnipeds, Thermoregulation

## Abstract

**Background:**

Pinnipeds have a thick blubber layer and may have difficulty maintaining their body temperature during hot weather when on land. The skin is the main thermoregulatory conduit which emits excessive body heat.

**Methods:**

Thorough evaluation of the skin histology in three pinniped species; the California sea lion-*Zalophus californianus*, the Pacific harbor seal-*Phoca vitulina richardsi*, and the Northern elephant seal-*Mirounga angustirostris*, was conducted to identify the presence, location and distribution of skin structures which contribute to thermoregulation. These structures included hair, adipose tissue, sweat glands, vasculature, and arteriovenous anastomoses (AVA). Thermal imaging was performed on live animals of the same species to correlate histological findings with thermal emission of the skin.

**Results:**

The presence and distribution of skin structures directly relates to emissivity of the skin in all three species. Emissivity of skin in phocids (Pacific harbor and Northern elephant seal*s*) follows a different pattern than skin in otariids (California sea lions). The flipper skin in phocids tends to be the most emissive region during hot weather and least emissive during cold weather. On the contrary in otariids, skin of the entire body has a tendency to be emissive during both hot and cold weather.

**Conclusion:**

Heat dissipation of the skin directly relates to the presence and distribution of skin structures in all three species. Different skin thermal dissipation patterns were observed in phocid versus otariid seals. Observed thermal patterns can be used for proper understanding of optimum thermal needs of seals housed in research facilities, rescue centers and zoo exhibits.

## Background

Thermoregulatory behavior in pinnipeds has been extensively described in the literature; specifically, changes in behaviors which are dependent on environmental air temperature [1-8]. Since air has poor thermal conductivity, marine mammals may encounter problems with overheating when on land; especially under direct sunlight [2,6,9,10]. Sea water on the other hand has 25 times greater thermal conductivity than air [11]; hence, overheating poses no actual danger when foraging in the sea. Northern elephant seals are pelagic animals spending most of their lives foraging in the open sea and only coming ashore during breeding and molting [12]. California sea lions; however, tend to live in close proximity to the coastline, seldom venturing more than 230 kilometers to the open sea [13]. It has been established, from a behavioral stand point, that otariids and phocids differ in the mode in which they dissipate body heat [1-3,5-8]. Otariids were described to use their flippers as the major site of heat loss [3,8,14]. Beentjes [8] described in great depth the behavioral thermoregulation in New Zealand sea lions. He indicated that postural adjustments were made in response to environmental temperature changes. Other authors, [15] stated that in California sea lions evaporative heat loss through flippers was ineffective. According to some authors Phocids do not pant or sweat and instead use other strategies to dissipate body heat [6,9]. These include behavioral changes such as flipping wet sand on their body, flipper waving or lying in a pool of water to increase conductive heat loss. They also employ the so called thermal windows or “hot spots” for thermoregulatory evaporation [1,5-7]. These windows are activated by selective redistribution of the blood into wet fur skin regions to enhance evaporative cooling [7].

Sweat, as an evaporative heat loss adaptation, is the most effective mode of heat exchange in mammals [11]. Generally, it is believed that phocids have poorly developed sweat glands and that otariids, on the contrary, have well developed sweat glands in the bare skin regions [16-18]. Furthermore, Ling [17] stated that Southern elephant seals have small single apocrine sweat glands present in their skin. Overall presence and distribution of sweat glands in different skin regions in otariids and phocids were not fully described in the literature.

The subcutaneous blubber layer is a major adaptation affecting thermoregulation in marine mammals [2,6,9,10]. The subcutaneous blubber has low thermal conductivity and significantly reduces heat transfer via conduction which helps to maintain core body temperature in the ocean, but impedes heat transfer on land [11].

Presence of hair is also a prominent factor in heat dissipation. Different hair distribution patterns exist among marine mammals [18,19]. Scheffer [19] summarized the presence of primary and secondary hair in pinnipeds. He stated that primary hairs are always retained. However, secondary hairs were omnipresent in fur seals, vestigial in some species such as the walrus and absent completely in monk and elephant seals. Additionally, hair density per skin area was reported to be highest in fur seals and lowest in walrus [19].

Arteriovenous anastomoses (AVA) are one of the key adaptations for thermoregulation in the seal’s skin. Tarasoff & Fisher [20] observed high vascularity in the hind flippers of otariid and phocid seals, strongly suggesting that hind flippers would have a prominent thermoregulatory role. Bryden & Molyneux [21] stated that AVA density in California sea lions was higher in the flippers than in other regions of the body.

The three pinniped species evaluated in this study range from the northern latitudes to temperate and tropical regions of the North American continent. Thermal stress due to hot weather conditions would occur in the southern extent of their range (Gulf of California), while thermal stress due to cold weather conditions would occur in the northern most extent of their habitat (Gulf of Alaska, Bering Sea).

This research was conducted to study histology of the skin and its structures in three different marine mammal species and to correlate those findings with predicted heat dissipation patterns by using infrared thermography.

## Methods

**Figure 1:**
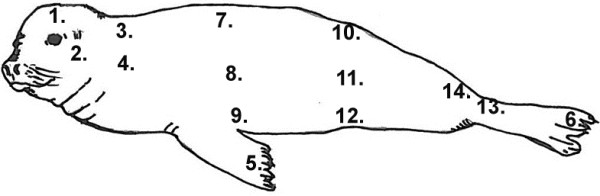
**Diagram showing sites where skin tissue samples were collected (see Table **1**).**

Skin tissue samples were collected from 8 pinniped specimens of both sexes (5 California sea lions-*Zalophus californianus*, 2 Pacific harbor seals-*Phoca vitulina richardsi*- and 1 Northern elephant seal-*Mirounga angustirostris*) ranging in age from 3 to 6 months. The animals were patients at the Marine Mammal Care Center Fort MacArthur, San Pedro, California and died due to various causes, other than skin infections. None of the selected animals were purposely euthanized for this project. Specimen acquisition was authorized by The United States Department of Commerce, National Oceanic and Atmospheric Administration, National Marine Fisheries Services. Institutional Animal Care Use Protocol was approved by The Western University of Health Sciences (#R09IACUC027). Full thickness skin samples and hypodermis from 14 different body sites from each animal (Figure 1) were excised and placed in 10% neutral buffered formalin (NBF) solution for fixation. Upon arrival to the laboratory samples, were trimmed to smaller sizes and kept in fresh NBF solution. Specimens were processed after 48 hours using standard histological technique [22]. Tissue samples were oriented using tissue markers and embedded in a way to expose all skin layers. Tissue blocks were sectioned at thicknesses of 5 to 7 microns using a rotary microtome and mounted on glass slides. Sections of each tissue sample were stained with Hematoxylin and Eosin, Masson’s Trichrome and Elastic stains [22]. A photographic camera, mounted on microscope (Nikon Eclipse E600) was used for histological examination and photography.

**Table 1:** Regional distribution of sweat glands and arteriovenous anastomoses in pinnipeds

**Skin region**	**Pacific harbor seal**		**Northern elephant seal**		**California sea lion**	
	**Sweat glands**	**AVA**	**Sweat glands**	**AVA**	**Sweat glands**	**AVA**
1. Head dorsal	+, not prominent	+	+, not prominent	-	+, not prominent	+
2. Head lateral	-	+	-	+	+	+
3. Cervical dorsal	+, not prominent	-	-	+	+	+
4. Cervical lateral	+, not prominent	-	-	-	+, prominent	+
5. Flipper fore	+, not prominent	+	+, not prominent	-	+, prominent	+
6. Flipper hind	+, not prominent	+	+, not prominent	+	+, prominent	+
7. Thoracic dorsal	-	**-**	-	-	+	-
8. Thoracic lateral	-	-	-	+	+	+
9. Thoracic ventral	+, not prominent	-	-	-	+	+
10. Abdomen dorsal	+, not prominent	-	-	-	+	+
11. Abdomen lateral	-	-	-	-	+	+
12. Abdomen ventral	-	+	-	-	+	+
13. Tail	+, not prominent	+	-	-	+, prominent	+
14. Gluteal dorsal	-	-	-	-	+	-

Sign ( + ) indicates presence. Sign (**–**) indicates absence. Sign (+), prominent) indicates a major presence, sign (+), not prominent) indicates scarce presence.

Infrared camera (Flir B series) was used to obtain full body thermographic images of all three species under different atmospheric conditions. The study was conducted at The Marine Mammal Care Center Fort MacArthur, San Pedro, CA from May to August 2010. All three species were thermographically assessed at environmental temperatures ranging from 16–30°C. Temperature and weather conditions for each recording session are presented in Table 2. Juvenile Northern elephant and Pacific harbor seals (3–8 month old) and adult California sea lions were used for thermography. The distance between the camera and the animals ranged between 1–3 meters. Imaging was performed under the sun and shade when the animals had dry and wet fur. The animals were held in small controlled areas at the facility and pictures were taken while ensuring photography did not cause physical or emotional distress to the animals. Animals chosen for thermography were in advanced stage of convalescence and ready to be released. Thermal images were analyzed using software.

**Table 2:** Dates of individual thermographic sessions, atmospheric conditions and timing of the sessions

**Date**	**Atmospheric conditions**	**Time of thermographic imaging**
05-18-2010	16-18°C, cold, cloudy day	11:00 am-1:00 pm
06-16-2010	20-22°C, with the sun coming out from the overcast clouds	1:00 pm-2:00 pm
08-15-2010	20-21°C, sunny with breeze	1:00 pm-2:00 pm
08-17-2010	25°C, sunny	1:00 pm-2:00 pm
09-02-2010	20°C, sunny	1:00 pm-2:00 pm
09-03-2010	20°C, sunny	1:00 pm-2:00 pm
09-27-2010	29-32°C, sunny and hot	1:00 pm-2:30 pm

## Results

### Epidermis

**Figure 2:**
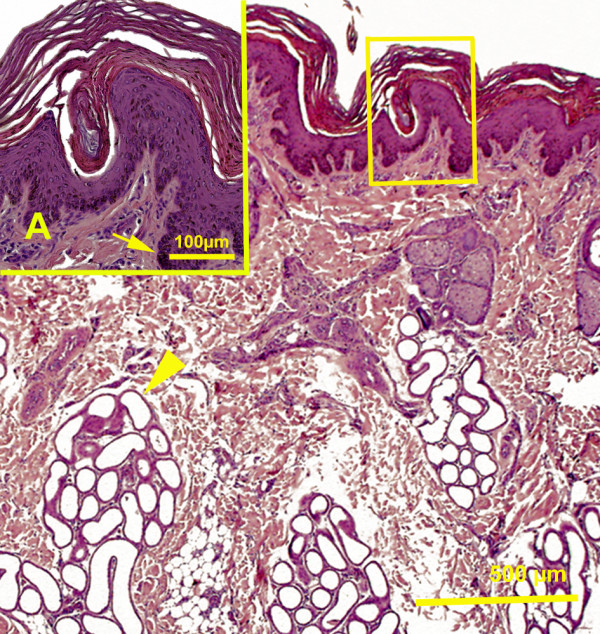
**California sea lion, hind flipper skin.** Magnification 4X. Hematoxylin and eosin stain. Sweat gland (arrowhead). Picture insert from the same slide **A**. Magnification 20 X. Stratum basale with prominent presence of melanin pigments (arrow).

Pinniped skin epithelium is stratified squamous (Figure 2) with variable degree of keratinization (moderate to high). The stratum corneum is recognizable in all skin segments; however, its thickness varied from region to region (Table 3). Stratum lucidum was absent in all three species in all body regions. Generally, more epithelial cell layers were present in ventral skin regions and the flippers. Conversely, in the head region, epidermal cell layers were less numerous. The presence of melanin pigment in the epidermis ranged from low to moderate and high in some skin regions. When present, melanocytes were located in the stratum basale of the epidermis in all species (Figure 2).

**Table 3:** Presence of structures/characteristics of the pinniped skin

**Characteristics**	**Harbor seal**	**Northern elephant seal**	**California sea lion**
1. Thin epidermis	+, head	+, head	+, head
2. Epidermis keratinization	+	+	+
3. Epidermal scale-like ridges	+	+	+
4. Thick epidermis	-	+	+ (vent. abd, tx, flippers)
5. Smooth muscle in dermis	+	+	+
6. Dermal veins w/t valves	+	+	+
7. Primary hair follicles	+, all the body	+, all the body	+, flippers bare ventrally
8. Dermal elastic fibers	+	+	+
9. Dermal collagen	+++	+++	+++
10. Wool hair (secondary hair follicles)	+ (head, vent. & dors. abd, vent., lat tx, glut., tail, flipper, neck, head)	-	+ (tx, vent & dors. abd, neck, glut, head)
11. Arrector pili	-	-	-
12. Sebaceous glands	+	+	+
13. Sweat glands	+ (not prominent)	+ (not prominent)	+ (prominent)
14. Brown adipose tissue	-	-	-
15. Hypodermal vessels	+	+	+
16. Hypodermal adipose tissue.	+++	+++	+++

Sign **+** indicates presence. Sign **–** indicates absence. Sign **+++** indicates a major presence. Vent. = ventral, dors. = dorsal, abd. = abdomen, tx. =thorax, glut. = gluteal.

In Northern elephant seals, the stratified squamous epithelium has 3 to 7 cell layers depending on the skin region; equally, in Pacific harbor seals the epidermis has 2–7 keratinocyte cell layers in different skin regions. The greatest variability in the thickness of epidermis was evident in California sea lions. It ranged from 2 cell layers in the head epidermis to 17 cell layers in the flippers (Figure 2). The presence of melanin pigment was moderate to high in Pacific harbor seals; whereas, it was low to moderate in Northern elephant seals and California sea lions.

In Northern elephant seals, the epidermis often created overlapping sharp ridges similar to the fish scales. These ridges usually sloped with hair direction. This characteristic feature was present in Harbor seals too, but was less evident.

### Dermis

In all species, the dermis was composed predominantly of collagenous fibers which are dispersed in multiple directions and infrequently fibroblasts and fibrocytes were observed. Bundles of collagen fibers were thicker and more prominent in the reticular zone (deep) of the dermis; while, the papillary zone had finer and more diffuse collagen fascicles. Orientation of the elastic fibers in the dermis was multidirectional (oblique, longitudinal, and transverse) with no apparent organization. Deeper segments of the dermis had much thicker elastic components. Arrector pili muscles were not observed in the dermis of any of these three species.

**Figure 3:**
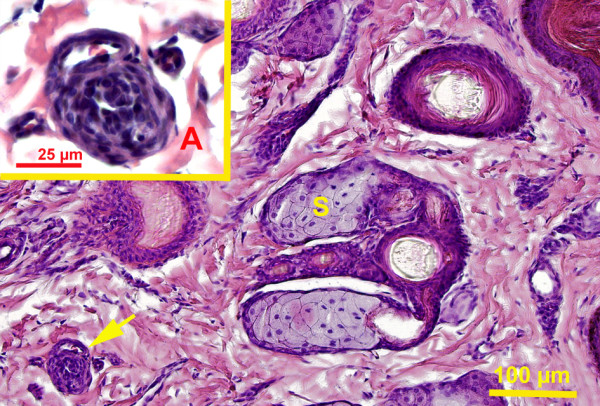
**Pacific harbor seal, hind flipper dermis.** Magnification 20 X. Hematoxylin and eosin stain. Sebaceous gland (S), indirect arteriovenous anastomosis, glomus body present (arrow). Picture insert from the same slide **A**. Magnification 100X. Indirect arteriovenous anastomosis.

**Figure 4:**
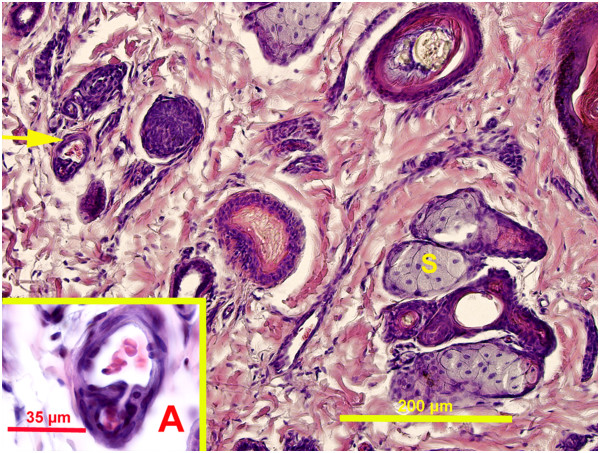
**Pacific harbor seal, hind flipper dermis.** Magnification 20 X. Hematoxylin and eosin stain. Sebaceous gland (S), direct arteriovenous anastomosis (arrow). Picture insert from the same slide **A**. Magnification 100X. Direct arteriovenous anastomosis.

**Figure 5:**
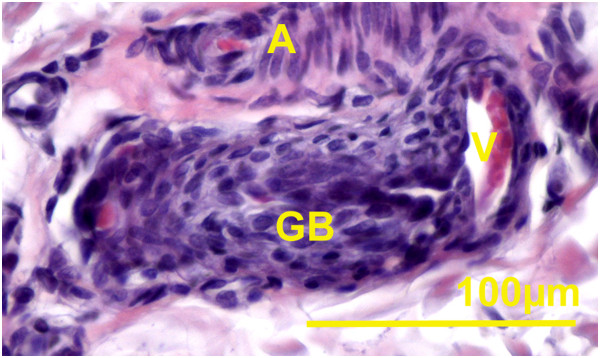
**Pacific harbor seal, hind flipper.** Indirect arteriovenous anastomosis. Magnification 100X. Hematoxylin and eosin stain. Arteriole (**A**), venule (V), glomus body with epitheloid cells (GB).

The papillary zone of the dermis had smaller vessels, blood and lymphatic capillaries, venules, and arterioles. The deeper layer of dermis generally contained larger blood vessels (veins and arteries). Large blood vessels were present at the junction between deep dermis and hypodermis. Large veins often had valves and were frequently accompanied by bundles of nerve fibers. In the flippers, dermis and hypodermis had a more pronounced vascular supply. Arteriovenous anastomoses (AVA) were observed close to the surface epithelium, deep to the hair follicles and dispersed between sweat glands. The majority of the AVAs were epithelioid in nature (Figures 3, 5). These AVA close to the surface epithelium developed into glomus body. Direct anastomoses with a small thickening of the arteriolar wall were also observed, but in much lesser number (Figure 4). Species specific and regional distributions of AVAs are summarized in Table 1.

The dermis had scattered multidirectional smooth muscle cell bundles. These bundles were present in larger quantities in the middle segment of the dermis in Northern elephant seals. In Pacific harbor seals smooth muscle cell bundles extended from the middle to the reticular zone of the dermis. Smooth muscle was absent at the papillary zone of the dermis.

**Figure 6:**
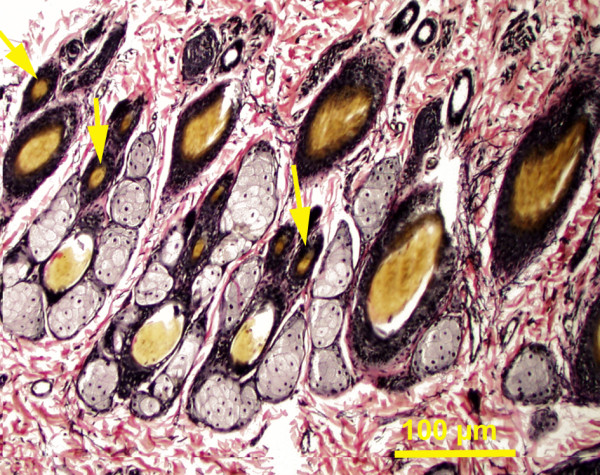
**Pacific harbor seal, lateral head skin region, dermis.** Magnification 10 X. Elastic stain. Secondary hair follicles (arrows).

Hair follicles were embedded in the dermis and each has sebaceous gland of varying size associated with it (Figures 3, 4, 6). At the base of the hair follicles, simple tubular sweat glands were observed occasionally in the head, fore and hind flippers in Northern elephant seals. In Pacific harbor seals, sweat glands were inconspicuous and occasionally observed in some skin segments in the neck, flippers and head. In California sea lions, the base of the hair follicles was always accompanied by a prominent simple coiled tubular apocrine sweat gland, particularly in the skin of the flippers and tail (Figure 2). In essence, sweat glands occupied most of the dermis in the skin of the flippers in this species. A few scattered sweat glands were found to be merocrine in nature. Myoepithelial cells were evident around these tubular sweat glands. The presence and distribution of sweat glands appeared to be species specific (Table 1 and 3).

In Pacific harbor seals, strands of collagenous connective tissue coursed through the dermis to attach to the base of the hair follicles. These strands of collagenous tissue serve as a framework for the passage of blood vessels and nerves to reach the upper dermis and the hair follicles.

Northern elephant and Pacific harbor seals generally lacked adipose tissue in the hypodermis of the flippers; whereas, in California sea lions hypodermal adipose tissue was always interspersed between large blood vessels.

### Hair

All three species have well developed primary (guard) hairs, while secondary (wool) hair presence varied greatly among them.

In the Pacific harbor seal, primary hair follicles were usually accompanied by 1–6 secondary hair follicles, in the ventral and dorsal abdomen, ventral and lateral thorax, gluteal region, tail, flippers, neck and head. Secondary hair follicles were more superficially located in the dermis than guard hair follicles (Figure 6). In California sea lions, secondary hair follicles were detected in the ventral and dorsal abdomen, thorax, lateral/dorsal neck and head regions and were absent in other regions of the body. The skin of the flippers in California sea lion was hairy dorsally and sparsely covered by hair ventrally (Figure 2). Northern elephant seals did not have secondary hair in any of the regions under study.

### Hypodermis

All three species had a well developed layer of subcutaneous adipose tissue which varied in thickness from region to region.

### Thermography results

#### Observations during pleasant to cool day

**Figure 7:**
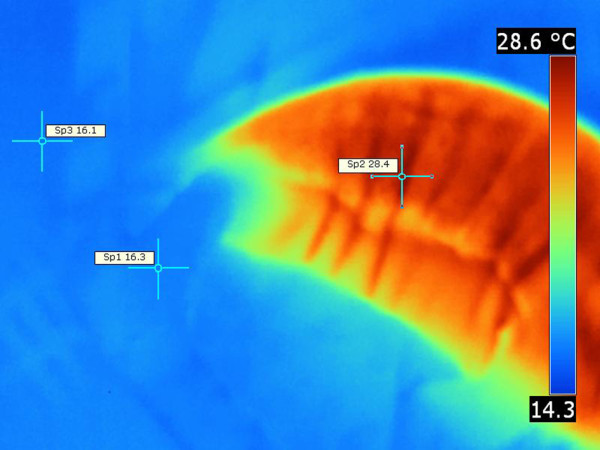
**Pacific harbor seal hind flipper, thermograph.** Note the hind flippers appear to emit very little heat, whereas, the body (trunk) of the seal is significantly more emissive. Environmental temperature was 17°C. T _max_ =28.4°C (Sp2), T _min_ =16.3°C (Sp2), ΔT = 12. 2°C, T _bkg_ = 16.1°C (Sp3).

**Figure 8:**
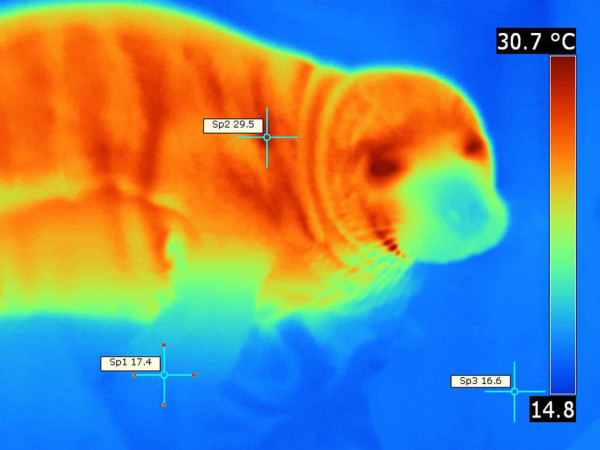
**Pacific harbor seal fore flipper and head thermograph.** Note that the fore flippers appear to emit very little heat together with the muzzle, whereas; the body (trunk), neck and the rest of the head is significantly hotter. Environmental temperature was 17°C. T _max_ =29.5°C (Sp2), T _min_ =17.4°C (Sp1), ΔT = 12.1, T _bkg_ =16.6°C (Sp3).

The flippers and muzzle were strikingly colder than the rest of the body in the Pacific harbor seal (Figures 7, 8). Folds of the truncal skin seemed to emit more heat, thus the highest temperature was recorded there. There was a notable difference [ΔT 12°C] between the trunk and the flipper temperatures (Figures 7, 8).

**Figure 9:**
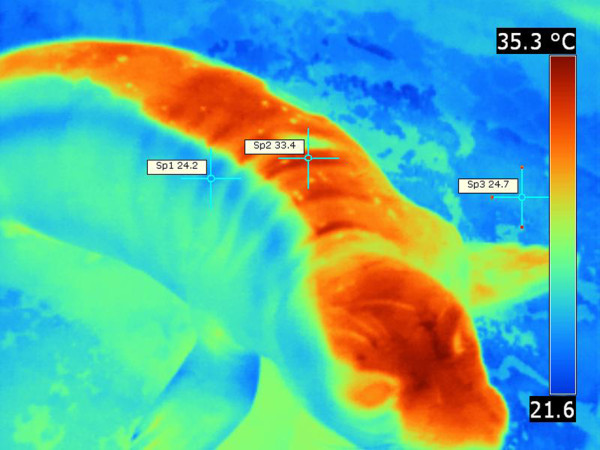
**Northern elephant seal, thermograph.** Wet region of the skin on right side of the body (blue) and dry skin region on the left side of the body (red). Note the clear demarcation between heat emitting dry skin region and rather cold wet skin region. Environmental temperature was 20.5-22.7°C. T _max_ =33.4°C (Sp2), T _min_ =24.2°C (Sp1), ΔT = 9.2°C, T _bkg_ =24.7°C (Sp3).

**Figure 10:**
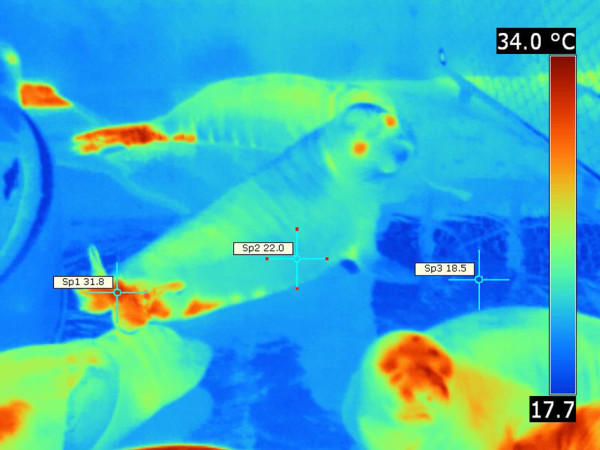
**Northern elephant seal, thermograph.** Please note “hot” hind flippers and “cold” fore flippers. Note the thermal windows or “hot” spots on the ventral abdomen. Environmental temperature was 20.5-22.7°C. T _max_ =31.8°C (Sp1), T _min_ =22°C (Sp2), ΔT = 9.8°C, T _bkg_ =18.5°C (Sp3).

Wet regions were exceptionally cool and dry regions emitted more heat in Northern elephant seals. During cool weather and in shade, wet skin segments appeared to emit less heat; while, dry segments of the skin appeared to emit more heat (Figure 9). In Northern elephant seals, in shade, the whole body had a lower surface temperature; however, the eyes and hind flippers showed increased heat emission. Consistently, thermal windows (described by Mauck et al. 2003) were observed on the hind flippers, while rarely observed on the fore flippers in this study (Figure 10). Thermal windows appeared even during relatively cold weather. They first appeared where the skin was bare, the blubber was thinnest and vascularity was most prominent. This is especially true for the hind flippers (Figure 10) and interdigital skin folds (webbings). Thermal windows sometimes appeared on the skin of the trunk as circumscribed circular “hot spots”, but were always preceded by the thermal windows on the hind flippers. Temperature differences between these thermal windows on the hind flipper and the rest of the body were ΔT 13–14°C.

**Figure 11:**
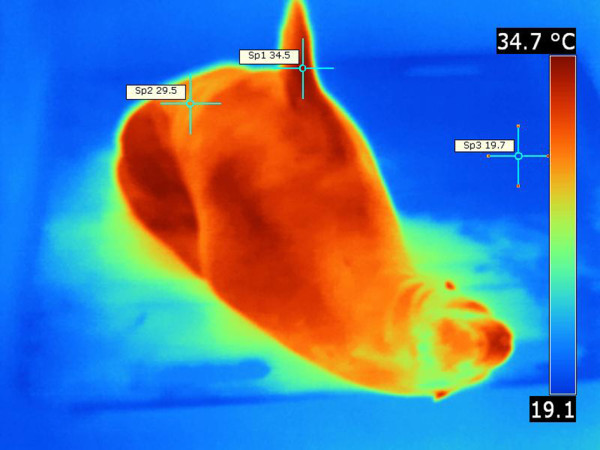
**California sea lion, thermograph.** Note diffuse skin region that dissipate heat. Note fore and hind flipper appeared hotter. Animal shows thermoregulatory behavior. Environmental temperature was 20°C. T _max_ =34.5°C (Sp1), T _min_ =29.5°C (Sp2), ΔT = 5°C, T _bkg_ =19.7°C (Sp3).

Even during relatively cool days, California sea lions dissipated body heat more diffusely throughout the entire skin. The eyes, head, nasal region, calvaria, dorsum of the trunk, and dorsum of the flippers emitted slightly more heat than other body regions (Figure 11). Bare skinned regions of the flippers were often more emissive than other body regions and higher surface temperatures were recorded (Figure 11). Ventral body regions were not prominent in heat dissipation. The “hot spot” pattern described in Northern elephant seal was not observed in California sea lions. However, in California sea lions even minimal locomotion produced changes in body surface temperatures. In summary, California sea lions did not present with distinct, well delineated thermal windows as in Northern elephant seals. On the contrary, their entire skin surface emitted heat (Figure 11).

#### Observations during warm to hot day

**Figure 12:**
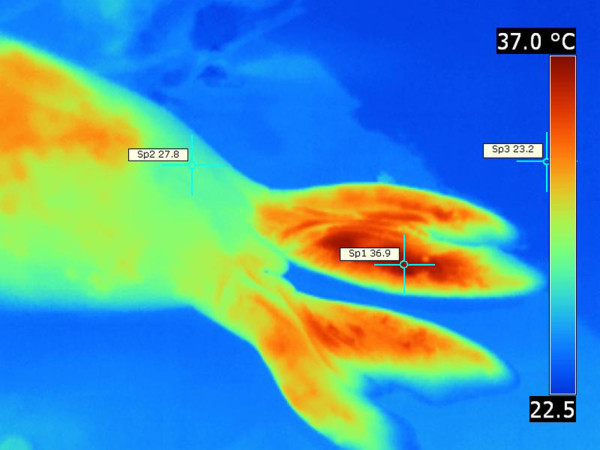
**Northern elephant seal, thermograph.** Note very emissive and hot hind flippers when compared with colder regions of the body. Environmental temperature was 29-32°C. T _max_ =36.9°C (Sp1), T _min_ =27.8°C (Sp2), ΔT = 9.1°C, T _bkg_ =23.2°C (Sp3).

In Northern elephant seals specific regions of the body emitted more heat than others. Most notable were the muzzle, eyes, dorsum of the head, neck and particularly hind flippers. During relatively hot atmospheric conditions (29-32°C), hind flippers were the site of the most intense heat emission and showed the highest surface temperature readings (Figure 12). Dorsally faced hyperpigmented skin membranes (webbings) which connect phalanges of adjacent digits on the hind flippers were associated with intense heat dissipation. These skin membranes were either completely devoid of hair, or the hair presence was scarce. Heat was dissipated from the flippers even during a relatively cool day; however, the intensity increased during hot weather. In addition to the hind flippers, the dorsum of the body “lights up” as either diffuse thermal windows or as a patchy array of “hot spots”. The appearance of thermal windows in the hind flippers always preceded their appearance in other body regions. There was a consistent temperature difference of ΔT 9.1°C between the hottest body region and the coldest body region in Northern elephant seals regardless of the environmental temperature at the time of recording.

Thermograms of California sea lions taken on a hot day revealed that skin of the entire body exhibited heat dissipating properties. The highest temperature readings were observed on the dorsum of the head, neck, thorax, and abdomen. Additionally, it was observed that free edges of the fore and hind flippers show intense heat dissipation. The edges of the flippers generally lacked hair while sweat glands were prominently present.

Pacific harbor seal thermal imaging was not performed as the animal was released prior to the onset of the hot weather.

## Discussion

The skin is a complex and dynamic organ that fulfills many functions, one of which is thermoregulation. Skin from 14 different body regions was histologically studied for the presence of skin structures. Surface temperature images captured on live seals using thermographic camera were assessed. Skin structures including sweat glands, hair, adipose tissue, arteriovenous anastomoses and vasculature play intricate roles in thermoregulation.

### Sweat glands and heat loss

Sweat glands are inconsistently present in true seals. When they are present they are usually scarce; on the contrary, sweat glands are prominent in fur seals and sea lions [15-18,23,24]. These observations were identical with our findings in California sea lions. Ling [17] stated that Southern elephant seals had small single apocrine sweat glands, which is also consistent with our occasional observations of sweat glands in Northern elephant and Pacific harbor seals. Evaporation is the most effective heat dissipation pathway; however, two species assessed (the Pacific harbor seal and Northern elephant seal) had small and infrequently present sweat glands all over the body. This indicates that evaporative cooling through sweating would not be an important mode of heat emission in these species. Northern elephant seals dissipated heat through thermal windows or “hot spots”. Mauck et al. [7] stated that these “hot spots” were present in seals due to evaporative heat loss. In our study, it was observed that they occurred also in dry skin regions, hence heat dissipation was more likely a result of radiative heat loss. Northern elephant seals, when on land, rely heavily on radiative cooling due to scarce presence of sweat glands. In California sea lions, sweat glands were prominent throughout the skin of the body and were especially dense in the flippers. Matsura & Whittow [15] tried to account for the percentage of heat loss in California sea lions via sweating. They stated that sweating accounted for 16 percent of the total heat loss, indicating that sweating was relatively ineffective in heat dissipation. In addition, they stated it only occurred on the bare surfaces of the flippers. In California sea lions in this study, during relatively hot weather, bare flippers were observed as the sites of strong heat loss. Limberger et al. [25] stated that flipper temperature in the Galapagos fur seal fluctuated due to the lack of hair cover; therefore the flippers were ideal for heat dissipation. On the other hand, Reidman [9] stated that marine mammals cannot sweat, but those species that spend significant time on land have retained the ability to sweat.

### Hair and heat loss

The distribution of hair varies among marine mammals [18,19]. Entirely aquatic marine mammals such as cetaceans have lost hair completely while sirenians have lost most of their hair. Some true seals and walrus have lost wool hair only; while fur seals, many true seals, and otters have kept the wool hair. This study confirmed scant presence of wool hair in the Pacific harbor seals and California sea lions. However, there was no wool hair observed in the Northern elephant seals. The fact that all three evaluated species do not have arrector pili muscles in the skin indicated that they seldom have problems with cold weather when on land. The presence of thick subcutaneous blubber layer which insulates seals from cold weather makes arrector pili muscle unnecessary. The lack of arrector pili muscles and the lack of the prominent wool hair suggest that air cannot be efficiently trapped in the fur. The absence in Northern elephant seals and sparse presence of wool hair in California sea lions would be conducive to radiative cooling during high ambient temperatures. McCafferty et al. [26] stated that the highest truncal temperature was evident only at sites where the Grey seal skin was dry after swimming. It seems that there is a distinct difference in heat loss between hairy and bare skin regions, where the bare skin regions often show greater heat loss and reveal thermal windows earlier than furred skin regions.

### Hypodermal adipose tissue (blubber) and heat loss

Hypodermal adipose tissue (blubber) of the seal’s creates a continuous layer, the panniculus adiposus. Many authors have speculated on thermoregulatory role of the hypodermal adipose tissue in seals [18,24,27,28]. Ling [18] affirmed that in adult Southern elephant seals the ventral body regions had a thicker blubber layer than the dorsal regions and in Northern elephant seals blubber in the thoracic region was ten times thicker than in the hind body region. Since blubber has poor thermal conductivity and is a good insulating substance, its presence would be sufficient to insulate the seals when swimming in relatively cold Pacific Ocean. The blubber might also impose a problem when these animals are on land during relatively warm periods of the year, thus impeding effective cooling mechanisms. One of the observations in this study was that thermal windows [7] appeared first in the areas of the body with little adipose tissue such as the flippers. The flippers presented as the coolest regions of the body when the animal was at relatively cool environmental temperature.

Areas where skin had a thick blubber layer, such as the dorsum, were generally showing lesser surface temperature when observed thermographically. Conversely, regions of the skin such as the flipper where blubber was thin showed higher surface temperatures. Paterson et al. [29] showed that flipper webbing in gray seals emitted more heat than the skin area at the level of the phalanges. Also, Ohata & Miller [4] observed that the interdigital webbing was a major site of heat dissipation in the Northern fur seal.

### Arteriovenous anastomoses and heat loss

Arteriovenous anastomoses are one of the key adaptations for thermoregulation. Tarasoff & Fisher [20] observed high vascularity in the hind flippers of otariid and phocid seals supporting their prominent thermoregulatory role. It is evident grossly, that large blood vessels are present within the skin webbings between the phalanges, exposing those vessels directly to bi-layered, thin and un-insulated skin. These sites tend to be the most heat dissipating regions of the body during hot weather and least heat emissive during the relatively cool weather. This suggests that AVA’s are redirecting blood flow from the core body to the flippers to dissipate heat when there is an increase in core body temperature due to physical activity or warm environment. In cool environment, the AVA’s would act to shunt blood towards core body to preserve body heat. Kuhn & Meyer [30] established that Eurasian otters dissipate heat through their feet, while manatees possess adaptations pertaining to heat conservation in their tail even though they live in warmer seas [31].

Bryden & Molyneux [21] quantitated the density of AVA per square centimeter in different pinniped species. They stated that in California sea lion and Northern fur seal AVA density was higher in flippers than in other regions of the body. Our study established that in California sea lions AVAs were present throughout the skin of the body, with the exception of the dorsal thoracic and gluteal regions. The general distribution of AVAs may play a role in diffuse heat dissipation pattern observed in California sea lions. Molyneux & Bryden [32] found that Weddell and Southern elephant seals do not differ in AVA density in the flipper versus the rest of the body. However, they stated that in phocid seals AVA density was many times greater when compared with otariids. Additionally, AVAs were observed closer to the surface epithelium in phocid seals [32,33]. Limberger et al. [25] stated that core body temperature in Galapagos sea lions was constant; however, flipper temperature fluctuated indicating that it might be the site of heat conservation in cold weather or heat dissipation in hot weather. In Pacific harbor seals, we detected AVAs in large numbers in the regions of the head, tail, ventral abdomen and particularly in the flippers. In Northern elephant seals we have detected AVAs in the head, neck, hind flipper and ventral abdominal skin. It seems that the fore flippers play a minor role in thermal regulation in Northern elephant seals, since they seldom showed thermal windows. They are generally smaller in size when compared with hind flippers, with a relatively small skin surface area. Fore flippers generally lack the large bare skin regions, which tend to be the sites of intense heat dissipation on the hind flippers. The majority of AVA observed in this study were epithelioid in nature, which is consistent with observations in other pinniped species [21,32,33]. Glomus body in AVA would probably act to dissipate body heat by shunting blood away from the capillary beds into the superficial veins where heat exchange takes place.

Darker fur/skin had higher surface temperatures when exposed to direct sunlight, for example in Zebra [34]; while, infrared heat loss of the pigmented skin was not higher than in sparsely pigmented skin [35,36]. It was observed in beaver that furless tail is used for thermoregulation [37]. Fish [38] also observed that hairless appendages such as the feet and tail in muskrats served as the main vehicle for heat dissipation on high ambient air temperatures. Irving et al. [39]stated that in fur seals bare flippers were heterothermous with the ΔT of 20°C, whereas body skin varied by ΔT 4°C. This is consistent with observations in this study; namely, flippers tended to be cooler during exposure to cold air and warmer than the air when exposed to warm environment. Øritsland & Ronald [40] stated that in Harp seal pups heat dissipates primarily via flippers and to a lesser extent through the skin of the rest of the body. This is consistent with the findings of this study in Northern elephant seals, namely during hot weather thermal windows first appeared on flippers and then in other regions of the trunk. In Harp seals during cold (water) environmental temperatures, heat was negligibly lost via the flippers [28] and this is consistent with our observation in the Pacific harbor seal and Northern elephant seal. Irving & Hart [41] observed that Harbor seal flippers are often cooler than the rest of the body; however, during warm weather they tend to be warmer than the rest of the body. This might be explained by the function of AVA which would shunt blood towards capillary beds when cold and redirect blood toward superficial veins during hot weather.

## Conclusion

The presence and distribution of skin structures directly relates to emissivity of the skin in all three species. Emissivity of skin in phocids (Pacific harbor and Northern elephant seals) follows a different pattern than skin in otariids (California sea lions). The flipper skin in phocids tends to be the most emissive region during hot weather and least emissive during cold weather. On the contrary in otariids, skin of the entire body has a tendency to be emissive during both hot and cold weather. Skin structures, we studied, will play an essential role in pinniped survival when on land.

## Competing interests

The authors do not have any competing interests; this is only an academic manuscript.

## Authors’ contributions

WAK initiated the project. JLR and LP did the necropsies and specimen acquisitions**.** JLR and WAK completed tissue processing, sectioning and staining. WAK, HS and LP did the thermographic imaging. WAK and HS did the write up of the manuscript**.** All authors read and approved final manuscript.
